# Marburg Virus in Fruit Bat, Kenya

**DOI:** 10.3201/eid1602.091269

**Published:** 2010-02

**Authors:** Ivan V. Kuzmin, Michael Niezgoda, Richard Franka, Bernard Agwanda, Wanda Markotter, Robert F. Breiman, Wun-Ju Shieh, Sherif R. Zaki, Charles E. Rupprecht

**Affiliations:** Centers for Disease Control and Prevention, Atlanta, Georgia, USA (I.V. Kuzmin, M. Niezgoda, R. Franka, W.-J. Shieh, S.R. Zaki, C.E. Rupprecht); National Museums of Kenya, Nairobi, Kenya (B. Agwanda); University of Pretoria, Pretoria, South Africa (W. Markotter); and Centers for Disease Control and Prevention–Kenya, Nairobi (R.F. Breiman)

**Keywords:** Lake Victoria Marburgvirus, Marburg virus, bats, Egyptian fruit bat, Rousettus aegyptiacus, zoonosis, Kenya, filovirus, viruses, letter

**To the Editor:** Lake Victoria Marburgvirus (MARV) causes severe hemorrhagic fever with a high case-fatality rate in humans. Index cases occurred in Europe during 1967 among laboratory workers who handled tissues and blood samples of nonhuman primates from Africa (*1*). Thereafter, MARV was reported throughout sub-Saharan Africa. Most outbreaks in humans were associated with visits to caves and mines (*2*–*6*). In Kenya, human cases of MARV infection were reported in 1980 and 1987; these occurred after visits to the Kitum Cave at Mount Elgon (*7*,*8*). MARV was detected in tissues of Egyptian fruit bats (*Rousettus aegyptiacus*) and other bat species from the Democratic Republic of Congo (DRC), Gabon, and Uganda (*3*–*6*).

We collected bats from across Kenya during June–July 2007 within the framework of the Global Disease Detection Program, which is dedicated to investigation of emerging pathogens. Collection protocols were approved by the National Museums of Kenya and by the Centers for Disease Control and Prevention (Atlanta, GA, USA). Blood, fecal and oral swab specimens, and selected tissue samples were collected from bats and stored on dry ice.

For MARV detection, total RNA was extracted from pooled or individual liver, spleen, and lung samples from 272 bats. Nested reverse transcription–PCR (RT-PCR) with primers specific for MARV nucleoprotein gene was performed as described (*5*). When a band of the expected size was detected after electrophoresis on an agarose gel, the RT-PCR product was sequenced. Laboratory cross-contamination was not a concern because no work with MARV had been conducted in the facility where the examination was performed.

MARV RNA was detected in pooled liver, spleen, and lung tissue of an apparently healthy, pregnant, female *R. aegyptiacus* bat obtained at Kitum Cave in July 2007 (Figure). A faint band was obtained only after nested RT-PCR, which suggests that the RNA load was limited. Attempts at virus isolation were not performed. Phylogenetic comparisons demonstrated that the virus (KE261, GenBank accession no. GQ499199) was relatively distant from previous isolates from Kenya (Musoke and Ravn). It was similar to viruses isolated from index cases in Europe in 1967 (Popp and Ci67). This lineage also contained virus 02DRC99, which was isolated from a human in the DRC in 1999 (Appendix Figure). MARV isolates obtained from bats and humans in Uganda in 2007 belong to distinct lineages (*6*) (Appendix Figure). Tissues of other bats, including 75 *R*. *aegyptiacus* (29 pregnant females) from Kitum Cave and neighboring Makingeni Cave, were negative for MARV RNA.

**Figure:**
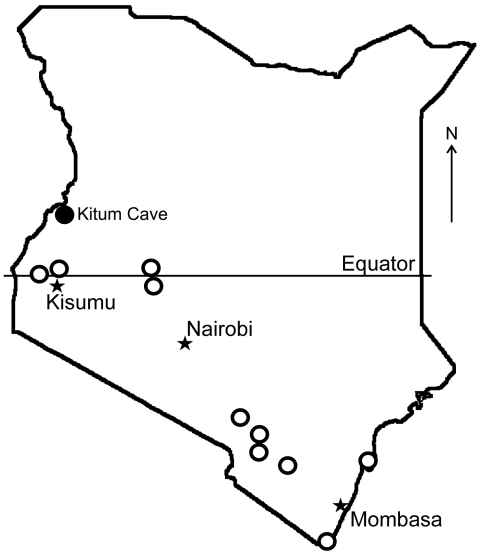
Bat collection sites (open circles) and location of Kitum Cave, Kenya, where Lake Victoria Marburgvirus was detected (solid circle).

Histopathologic examination of liver of the infected bat showed no lesions that could be ascribed to MARV infection, and no MARV antigens were detected by immunohistochemical analysis. Other tissues were not examined.

Our results are similar to those reported from Gabon and the DRC, where MARV RNA was detected in tissues of 1.4% and 3.1% of *R*. *aegyptiacus* bats, respectively, with negative isolation attempts (*3*,*5*). A higher prevalence (5.1%) was detected in *R*. *aegyptiacus* bats from Uganda in 2007, where several MARV isolates were obtained from bats with high virus loads (*6*). In the DRC, MARV RNA was also detected in insectivorous bats, including 3.0% of *Miniopterus inflatus* and 3.6% of *Rhinolophus elocuens* (*3*,*5*). However, in Uganda, MARV RNA was detected in only 1 (0.2%) of 609 insectivorous bats (*Hipposideros* spp.) (*6*).

To date, bats are the only wild mammals, besides nonhuman primates, in which filoviruses have been detected. Whether bats serve as principal reservoir hosts for filoviruses is unclear. The pathogenesis and clinic manifestation of filovirus infection in bats are unknown. Colonies of *R*. *aegyptiacus* bats in caves often consist of thousands of bats. The opportunity for conspecific exposure rates in such colonies is high. Therefore, bat populations should have a high seroprevalence rate for these viruses. For example, seroprevalence to lyssaviruses in some bat species that live in colonies was reported as high as 60%–70% (*9*). In contrast, seroprevalence of MARV-neutralizing antibodies in colonies of *R*. *aegyptiacus* bats in which PCR-positive bats were collected was only 12% (*5*) or as low as 2.4% (*6*). This low seroprevalence may be interpreted as a result of a limited spillover of MARV into bats from another source.

The association of human cases of MARV with visiting caves often inhabited by *R*. *aegyptiacus* and other bat species is obvious (*3*,*5*,*6*). This association was reinforced by MARV infection in tourists who visited caves in Uganda (*4*,*10*). For Kenya, our finding is consistent with reported human cases tentatively associated with visiting of Kitum Cave (*7*,*8*). We do not know if MARV has persisted in this area continuously or has reemerged sporadically. Kitum Cave and other similar caves are easily accessible and frequently visited by tourists and local persons. The likelihood of MARV spillover into humans is presently limited. However, because transmission mechanisms and sources of spillover infections are unknown, public awareness must be increased and health authorities informed about the presence of MARV.

## Supplementary Material

Appendix Figure Phylogenetic position of Lake Victoria Marburgvirus isolate KE261 (in boldface) among other Marburg viruses, based on the 400-nt fragment of the nucleoprotein gene. GenBank accession numbers, sequence names, and origins (in parentheses) are indicated. Bootstrap support was calculated for 1,000 replicates. Scale bar indicates nucleotide substitutions per site. DRC, Democratic Republic of Congo.
