# Supplementary figures and images for: Marburg Virus in Fruit Bat, Kenya
Source: Emerg Infect Dis. 2010 Feb;16(2):352–4. doi: 10.3201/eid1602.091269 (PMC2958024; doi:10.3201/eid1602.091269)

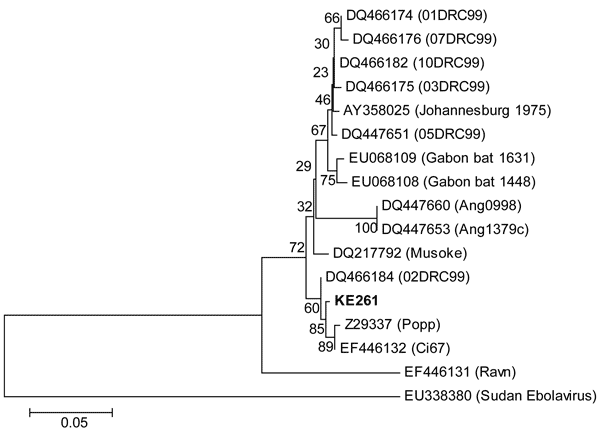

Supplement: Appendix Figure — Phylogenetic position of Lake Victoria Marburgvirus isolate KE261 (in boldface) among other Marburg viruses, based on the 400-nt fragment of the nucleoprotein gene. GenBank accession numbers, sequence names, and origins (in parentheses) are indicated. Bootstrap support was calculated for 1,000 replicates. Scale bar indicates nucleotide substitutions per site. DRC, Democratic Republic of Congo. [file 09-1269_appF-s1.gif]
